# Bis(propan-2-yl) [(2*S*,3*S*)-2-hydr­oxy-3-nitro­butan-2-yl]phospho­nate

**DOI:** 10.1107/S1600536809052428

**Published:** 2009-12-12

**Authors:** Tanmay Mandal, Sampak Samanta, Grant A. Broker, Cong-Gui Zhao, Edward R. T. Tiekink

**Affiliations:** a Department of Chemistry, University of Texas at San Antonio, One UTSA Circle, San Antonio, Texas 78249-0698, USA; b Department of Chemistry, University of Malaya, 50603 Kuala Lumpur, Malaysia

## Abstract

In the title compound, C_10_H_22_NO_6_P, a staggered conformation is found when the mol­ecule is viewed down the central P—C bond, with the oxo and hydr­oxy groups *gauche* to each other. The crystal structure features supra­molecular chains of helical topology propagating along the *b* axis, mediated by O—H⋯O hydrogen bonds.

## Related literature

For background to the enanti­oselective nitro­aldol reaction of α-ketophospho­nates and nitro­methane and for the synthesis, see: Mandal *et al.* (2007 ▶).
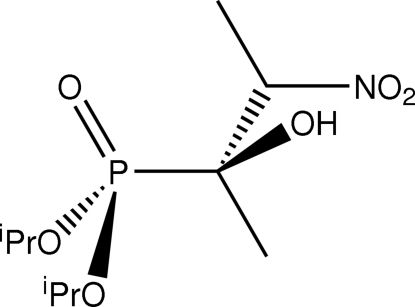


## Experimental

### 

#### Crystal data

C_10_H_22_NO_6_P
*M* *_r_* = 283.26
Orthorhombic, 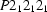

*a* = 7.8620 (16) Å
*b* = 11.369 (2) Å
*c* = 16.920 (3) Å
*V* = 1512.4 (5) Å^3^
*Z* = 4
Mo *K*α radiation
μ = 0.20 mm^−1^
*T* = 173 K
0.32 × 0.10 × 0.05 mm

#### Data collection

Rigaku AFC12/SATURN724 diffractometer
Absorption correction: multi-scan (*ABSCOR*; Higashi, 1995 ▶) *T*_min_ = 0.884, *T*_max_ = 1
13441 measured reflections
3072 independent reflections
3020 reflections with *I* > 2σ(*I*)
*R*_int_ = 0.089
Standard reflections: 0

#### Refinement

*R*[*F* ^2^ > 2σ(*F* ^2^)] = 0.040
*wR*(*F* ^2^) = 0.105
*S* = 1.07
3072 reflections
166 parameters
1 restraint
H-atom parameters constrained
Δρ_max_ = 0.19 e Å^−3^
Δρ_min_ = −0.25 e Å^−3^
Absolute structure: Flack (1983 ▶), 1272 Friedel pairs
Flack parameter: 0.05 (11)

### 

Data collection: *CrystalClear* (Rigaku/MSC, 2005 ▶); cell refinement: *CrystalClear*; data reduction: *CrystalClear*; program(s) used to solve structure: *SHELXS97* (Sheldrick, 2008 ▶); program(s) used to refine structure: *SHELXL97* (Sheldrick, 2008 ▶); molecular graphics: *DIAMOND* (Brandenburg, 2006 ▶); software used to prepare material for publication: *publCIF* (Westrip, 2009 ▶).

## Supplementary Material

Crystal structure: contains datablocks global, I. DOI: 10.1107/S1600536809052428/hb5269sup1.cif

Structure factors: contains datablocks I. DOI: 10.1107/S1600536809052428/hb5269Isup2.hkl

Additional supplementary materials:  crystallographic information; 3D view; checkCIF report

## Figures and Tables

**Table 1:** Hydrogen-bond geometry (Å, °)

*D*—H⋯*A*	*D*—H	H⋯*A*	*D*⋯*A*	*D*—H⋯*A*
O4—H4o⋯O1^i^	0.84	1.90	2.7289 (19)	172

Symmetry code: (i) 
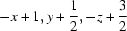
.

**Fig. 1.**
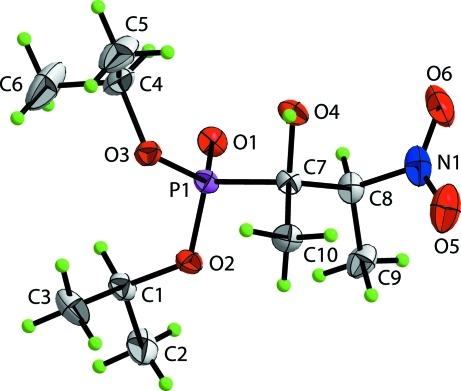
Molecular structure of (I), showing displacement ellipsoids at the 35% probability level.

**Fig. 2.**
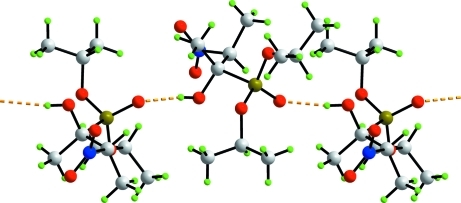
Supramolecular chain along the b axis in (I) mediated by O–H···O (orange dashed lines) hydrogen bonding. Colour scheme: P, olive; O, red; N, blue; C, grey; and H, green.

## supplementary crystallographic information

### Comment

The title compound, (I), was investigated as a part of previous studies on the enantioselective nitroaldol reaction of α-ketophosphonates and nitromethane for the synthesis of optically active α-hydroxy-β-nitrophosphonates (Mandal *et al.*, 2007). The crystal structure analysis of (I), Fig. 1, shows a staggered conformation when the molecule is viewed down the P–C7 axis in which the oxo and hydroxy groups are *gauche* to each other. The presence of O–H···O hydrogen bonding formed between the hydroxy-O4—H and O═P atoms leads to the formation of supramolecular chains along the *b* axis, Fig. 2 and Table 1.

### Experimental

The title compound was prepared as described in the literature (Mandal *et al.*, 2007).

### Refinement

The C-bound H atoms were geometrically placed (C—H = 0.98–1.00 Å) and refined as riding with *U*_iso_(H) = 1.2–1.5*U*_eq_(C). The methyl H-atoms were rotated to fit the electron density. The O–H H atom was located from a difference map and refined with O–H = 0.840±0.001 Å, and with *U*_iso_(H) = 1.5*U*_eq_(O).

### Crystal data

**Table:**

C_10_H_22_NO_6_P	*F*(000) = 608
*M**_r_* = 283.26	*D*_x_ = 1.244 Mg m^−^^3^
Orthorhombic, *P*2_1_2_1_2_1_	Mo *K*α radiation, λ = 0.71073 Å
Hall symbol: P 2ac 2ab	Cell parameters from 2308 reflections
*a* = 7.8620 (16) Å	θ = 4.0–30.1°
*b* = 11.369 (2) Å	µ = 0.20 mm^−^^1^
*c* = 16.920 (3) Å	*T* = 173 K
*V* = 1512.4 (5) Å^3^	Block, pale-yellow
*Z* = 4	0.32 × 0.10 × 0.05 mm

### Data collection

**Table:**

Rigaku AFC12K/SATURN724 diffractometer	3072 independent reflections
Radiation source: fine-focus sealed tube	3020 reflections with *I* > 2σ(*I*)
graphite	*R*_int_ = 0.089
ω scans	θ_max_ = 26.5°, θ_min_ = 4.0°
Absorption correction: multi-scan (*ABSCOR*; Higashi, 1995)	*h* = −9→8
*T*_min_ = 0.884, *T*_max_ = 1	*k* = −13→14
13441 measured reflections	*l* = −21→20

### Refinement

**Table:**

Refinement on *F*^2^	Secondary atom site location: difference Fourier map
Least-squares matrix: full	Hydrogen site location: inferred from neighbouring sites
*R*[*F*^2^ > 2σ(*F*^2^)] = 0.040	H-atom parameters constrained
*wR*(*F*^2^) = 0.105	*w* = 1/[σ^2^(*F*_o_^2^) + (0.0497*P*)^2^ + 0.2822*P*] where *P* = (*F*_o_^2^ + 2*F*_c_^2^)/3
*S* = 1.07	(Δ/σ)_max_ = 0.001
3072 reflections	Δρ_max_ = 0.19 e Å^−^^3^
166 parameters	Δρ_min_ = −0.25 e Å^−^^3^
1 restraint	Absolute structure: Flack (1983), 1272 Friedel pairs
Primary atom site location: structure-invariant direct methods	Flack parameter: 0.05 (11)

### Special details

**Table:**

Geometry. All e.s.d.'s (except the e.s.d. in the dihedral angle between two l.s. planes) are estimated using the full covariance matrix. The cell e.s.d.'s are taken into account individually in the estimation of e.s.d.'s in distances, angles and torsion angles; correlations between e.s.d.'s in cell parameters are only used when they are defined by crystal symmetry. An approximate (isotropic) treatment of cell e.s.d.'s is used for estimating e.s.d.'s involving l.s. planes.
Refinement. Refinement of *F*^2^ against ALL reflections. The weighted *R*-factor *wR* and goodness of fit *S* are based on *F*^2^, conventional *R*-factors *R* are based on *F*, with *F* set to zero for negative *F*^2^. The threshold expression of *F*^2^ > σ(*F*^2^) is used only for calculating *R*-factors(gt) *etc*. and is not relevant to the choice of reflections for refinement. *R*-factors based on *F*^2^ are statistically about twice as large as those based on *F*, and *R*- factors based on ALL data will be even larger.

### Fractional atomic coordinates and isotropic or equivalent isotropic displacement parameters (Å^2^)

**Table:**

	*x*	*y*	*z*	*U*_iso_*/*U*_eq_	
P1	0.63065 (6)	0.29509 (4)	0.74005 (3)	0.03236 (14)	
O1	0.51586 (18)	0.19464 (11)	0.75316 (9)	0.0416 (3)	
O2	0.80539 (18)	0.26553 (13)	0.70112 (9)	0.0415 (3)	
O3	0.67933 (17)	0.36408 (13)	0.81677 (8)	0.0383 (3)	
O4	0.41385 (17)	0.46364 (12)	0.71762 (9)	0.0400 (3)	
H4O	0.4448	0.5337	0.7241	0.060*	
O5	0.3878 (4)	0.49342 (19)	0.51776 (13)	0.0848 (7)	
O6	0.1751 (3)	0.4227 (2)	0.58457 (14)	0.0861 (7)	
N1	0.3262 (3)	0.4256 (2)	0.56573 (14)	0.0608 (6)	
C1	0.9313 (3)	0.18894 (18)	0.74033 (13)	0.0438 (5)	
H1	0.8736	0.1412	0.7821	0.053*	
C2	0.9985 (4)	0.1089 (3)	0.67718 (17)	0.0706 (8)	
H2A	0.9051	0.0617	0.6556	0.106*	
H2B	1.0849	0.0567	0.6998	0.106*	
H2C	1.0495	0.1560	0.6348	0.106*	
C3	1.0653 (3)	0.2668 (2)	0.7782 (2)	0.0656 (8)	
H3A	1.0121	0.3162	0.8187	0.098*	
H3B	1.1173	0.3168	0.7377	0.098*	
H3C	1.1530	0.2175	0.8026	0.098*	
C4	0.5800 (3)	0.3614 (2)	0.89018 (13)	0.0551 (6)	
H4	0.4659	0.3254	0.8803	0.066*	
C5	0.5601 (6)	0.4848 (3)	0.91703 (18)	0.0879 (11)	
H5A	0.4970	0.5295	0.8771	0.132*	
H5B	0.6726	0.5202	0.9246	0.132*	
H5C	0.4976	0.4862	0.9671	0.132*	
C6	0.6764 (7)	0.2898 (3)	0.94900 (18)	0.1056 (15)	
H6A	0.6874	0.2089	0.9296	0.158*	
H6B	0.6152	0.2896	0.9995	0.158*	
H6C	0.7897	0.3238	0.9565	0.158*	
C7	0.5409 (2)	0.40550 (16)	0.67209 (11)	0.0336 (4)	
C8	0.4436 (3)	0.33911 (18)	0.60636 (12)	0.0419 (5)	
H8	0.3722	0.2770	0.6318	0.050*	
C9	0.5556 (4)	0.2804 (2)	0.54440 (13)	0.0558 (6)	
H9A	0.4838	0.2406	0.5053	0.084*	
H9B	0.6299	0.2227	0.5700	0.084*	
H9C	0.6251	0.3401	0.5179	0.084*	
C10	0.6767 (3)	0.49079 (18)	0.64308 (14)	0.0430 (5)	
H10A	0.7312	0.5287	0.6885	0.065*	
H10B	0.6239	0.5509	0.6095	0.065*	
H10C	0.7623	0.4477	0.6125	0.065*	

### Atomic displacement parameters (Å^2^)

**Table:**

	*U*^11^	*U*^22^	*U*^33^	*U*^12^	*U*^13^	*U*^23^
P1	0.0303 (2)	0.0284 (2)	0.0384 (2)	0.00159 (18)	0.00078 (17)	0.00062 (19)
O1	0.0432 (7)	0.0284 (6)	0.0532 (8)	−0.0023 (5)	−0.0002 (6)	0.0044 (7)
O2	0.0357 (7)	0.0454 (8)	0.0435 (7)	0.0140 (6)	0.0051 (6)	0.0031 (6)
O3	0.0365 (7)	0.0426 (7)	0.0359 (7)	−0.0048 (6)	0.0024 (5)	−0.0017 (6)
O4	0.0342 (7)	0.0316 (7)	0.0541 (8)	0.0031 (6)	0.0021 (6)	−0.0080 (6)
O5	0.1205 (19)	0.0631 (12)	0.0708 (13)	0.0063 (14)	−0.0288 (14)	0.0171 (10)
O6	0.0593 (13)	0.1078 (17)	0.0912 (16)	0.0212 (13)	−0.0342 (12)	−0.0093 (14)
N1	0.0736 (16)	0.0513 (12)	0.0576 (12)	0.0103 (11)	−0.0261 (11)	−0.0073 (11)
C1	0.0408 (10)	0.0399 (11)	0.0507 (11)	0.0124 (8)	−0.0022 (9)	0.0007 (10)
C2	0.0768 (19)	0.0698 (17)	0.0650 (16)	0.0410 (16)	−0.0121 (14)	−0.0150 (14)
C3	0.0423 (12)	0.0584 (15)	0.096 (2)	0.0113 (11)	−0.0130 (13)	−0.0103 (14)
C4	0.0576 (14)	0.0725 (16)	0.0351 (10)	−0.0157 (12)	0.0104 (9)	−0.0010 (10)
C5	0.128 (3)	0.085 (2)	0.0507 (15)	0.038 (2)	0.0216 (17)	−0.0070 (15)
C6	0.189 (5)	0.081 (2)	0.0471 (15)	0.029 (3)	0.004 (2)	0.0180 (16)
C7	0.0344 (9)	0.0283 (8)	0.0383 (9)	0.0022 (7)	−0.0010 (8)	−0.0021 (8)
C8	0.0498 (12)	0.0332 (9)	0.0428 (10)	0.0031 (9)	−0.0088 (9)	−0.0032 (9)
C9	0.0772 (17)	0.0502 (13)	0.0399 (11)	0.0049 (13)	0.0000 (11)	−0.0097 (10)
C10	0.0451 (11)	0.0334 (10)	0.0506 (12)	−0.0024 (9)	0.0042 (9)	0.0032 (9)

### Geometric parameters (Å, °)

**Table:**

P1—O1	1.4723 (14)	C4—C5	1.483 (4)
P1—O2	1.5602 (14)	C4—C6	1.492 (4)
P1—O3	1.5643 (15)	C4—H4	1.0000
P1—C7	1.8428 (19)	C5—H5A	0.9800
O2—C1	1.476 (2)	C5—H5B	0.9800
O3—C4	1.467 (2)	C5—H5C	0.9800
O4—C7	1.424 (2)	C6—H6A	0.9800
O4—H4O	0.8400	C6—H6B	0.9800
O5—N1	1.219 (3)	C6—H6C	0.9800
O6—N1	1.230 (4)	C7—C10	1.524 (3)
N1—C8	1.514 (3)	C7—C8	1.546 (3)
C1—C2	1.500 (3)	C8—C9	1.523 (3)
C1—C3	1.517 (3)	C8—H8	1.0000
C1—H1	1.0000	C9—H9A	0.9800
C2—H2A	0.9800	C9—H9B	0.9800
C2—H2B	0.9800	C9—H9C	0.9800
C2—H2C	0.9800	C10—H10A	0.9800
C3—H3A	0.9800	C10—H10B	0.9800
C3—H3B	0.9800	C10—H10C	0.9800
C3—H3C	0.9800		
			
O1—P1—O2	115.86 (9)	C4—C5—H5A	109.5
O1—P1—O3	114.44 (9)	C4—C5—H5B	109.5
O2—P1—O3	104.06 (8)	H5A—C5—H5B	109.5
O1—P1—C7	112.80 (9)	C4—C5—H5C	109.5
O2—P1—C7	102.75 (8)	H5A—C5—H5C	109.5
O3—P1—C7	105.67 (8)	H5B—C5—H5C	109.5
C1—O2—P1	121.86 (13)	C4—C6—H6A	109.5
C4—O3—P1	124.17 (14)	C4—C6—H6B	109.5
C7—O4—H4O	108.0	H6A—C6—H6B	109.5
O5—N1—O6	124.9 (3)	C4—C6—H6C	109.5
O5—N1—C8	118.1 (2)	H6A—C6—H6C	109.5
O6—N1—C8	117.0 (2)	H6B—C6—H6C	109.5
O2—C1—C2	105.91 (18)	O4—C7—C10	111.74 (15)
O2—C1—C3	108.14 (17)	O4—C7—C8	105.60 (16)
C2—C1—C3	114.2 (2)	C10—C7—C8	115.18 (18)
O2—C1—H1	109.5	O4—C7—P1	104.31 (12)
C2—C1—H1	109.5	C10—C7—P1	111.47 (14)
C3—C1—H1	109.5	C8—C7—P1	107.80 (13)
C1—C2—H2A	109.5	N1—C8—C9	108.94 (19)
C1—C2—H2B	109.5	N1—C8—C7	108.11 (16)
H2A—C2—H2B	109.5	C9—C8—C7	115.0 (2)
C1—C2—H2C	109.5	N1—C8—H8	108.2
H2A—C2—H2C	109.5	C9—C8—H8	108.2
H2B—C2—H2C	109.5	C7—C8—H8	108.2
C1—C3—H3A	109.5	C8—C9—H9A	109.5
C1—C3—H3B	109.5	C8—C9—H9B	109.5
H3A—C3—H3B	109.5	H9A—C9—H9B	109.5
C1—C3—H3C	109.5	C8—C9—H9C	109.5
H3A—C3—H3C	109.5	H9A—C9—H9C	109.5
H3B—C3—H3C	109.5	H9B—C9—H9C	109.5
O3—C4—C5	107.2 (2)	C7—C10—H10A	109.5
O3—C4—C6	107.8 (2)	C7—C10—H10B	109.5
C5—C4—C6	111.4 (3)	H10A—C10—H10B	109.5
O3—C4—H4	110.1	C7—C10—H10C	109.5
C5—C4—H4	110.1	H10A—C10—H10C	109.5
C6—C4—H4	110.1	H10B—C10—H10C	109.5
			
O1—P1—O2—C1	−63.20 (17)	O3—P1—C7—C10	68.93 (15)
O3—P1—O2—C1	63.34 (17)	O1—P1—C7—C8	−37.99 (17)
C7—P1—O2—C1	173.35 (15)	O2—P1—C7—C8	87.48 (15)
O1—P1—O3—C4	−21.1 (2)	O3—P1—C7—C8	−163.73 (13)
O2—P1—O3—C4	−148.58 (17)	O5—N1—C8—C9	−47.5 (3)
C7—P1—O3—C4	103.58 (19)	O6—N1—C8—C9	133.2 (2)
P1—O2—C1—C2	136.98 (19)	O5—N1—C8—C7	78.1 (3)
P1—O2—C1—C3	−100.2 (2)	O6—N1—C8—C7	−101.1 (3)
P1—O3—C4—C5	−132.8 (2)	O4—C7—C8—N1	51.7 (2)
P1—O3—C4—C6	107.1 (3)	C10—C7—C8—N1	−72.1 (2)
O1—P1—C7—O4	73.94 (14)	P1—C7—C8—N1	162.77 (16)
O2—P1—C7—O4	−160.59 (12)	O4—C7—C8—C9	173.70 (17)
O3—P1—C7—O4	−51.80 (13)	C10—C7—C8—C9	49.9 (2)
O1—P1—C7—C10	−165.33 (14)	P1—C7—C8—C9	−75.2 (2)
O2—P1—C7—C10	−39.85 (16)		

### Hydrogen-bond geometry (Å, °)

**Table:**

*D*—H···*A*	*D*—H	H···*A*	*D*···*A*	*D*—H···*A*
O4—H4o···O1^i^	0.84	1.90	2.7289 (19)	172

Symmetry codes: (i) −*x*+1, *y*+1/2, −*z*+3/2.
